# Could master protocols be adapted for effectiveness-implementation hybrid studies?

**DOI:** 10.1186/s12874-025-02684-1

**Published:** 2025-11-18

**Authors:** Justin J. Chapman, Taren Massey-Swindle, Urska Arnautovska, Ingrid J. Hickman, Amanda J. Wheeler, Dan Siskind, Jeroen Deenik, Robert S. Ware, James A. Roberts, Yong Yi Lee, Alyssa Milton, Wolfgang Marx, Stephen J. Wood, Zoe Rutherford, Catherine Kaylor-Hughes, Mike Trott, Co-design and Research Translation Alliance in Mental Health (CoRTA-MH), The ALIVE National Centre for Mental Health Research Translation’s Implementation and Translation Network, Ravi Iyer

**Affiliations:** 1 Griffith University, Centre for Mental Health, School of Pharmacy and Medical Science, 170 Kessels Road, Nathan, Brisbane, QLD 4111 Australia; 2 Addiction and Mental Health Service, Metro South Health, Brisbane, QLD Australia; 3 Equally Well, School of Business, Charles Sturt University, Bathurst, NSW Australia; 4 The ALIVE National Centre for Mental Health Research Translation, University of Melbourne, Melbourne, VIC Australia; 5 Department of Pediatrics, University of Arkansas for Medical Sciences, Little Rock, AR USA; 6 Arkansas Children’s Nutrition Center, Little Rock, AR USA; 7 Arkansas Children’s Research Institute, Little Rock, AR USA; 8 Faculty of Medicine, University of Queensland, Brisbane, QLD Australia; 9 Queensland Centre for Mental Health Research, Brisbane, QLD Australia; 10 ULTRA Team, The University of Queensland Clinical Trials Capability, Centre for Clinical Research, Herston, Brisbane, QLD Australia; 11 Faculty of Medical and Health Sciences, Auckland University, Auckland, New Zealand; 12 GGz Centraal, Utrechtseweg 266, Amersfoort, 3818EW The Netherlands; 13 Mental Health and Neuroscience Research Institute, Maastricht University, PO Box 616, 5200MD Maastricht, The Netherlands; 14 Griffith Biostatistics Unit, Griffith University, Brisbane, QLD Australia; 15 QIMR Berghofer, Brisbane, QLD Australia; 16 Monash University Health Economics Group, School of Public Health and Preventive Medicine, Monash University, Melbourne, VIC Australia; 17 School of Public Health, The University of Queensland, Brisbane, QLD Australia; 18 Faculty of Medicine and Health, The University of Sydney, Sydney, NSW Australia; 19 ARC Centre of Excellence for Children and Families Over the Life Course, Sydney, NSW Australia; 20 Brain and Mind Centre, The University of Sydney, Sydney, NSW Australia; 21 Deakin University, IMPACT - the Institute for Mental and Physical Health and Clinical Translation, Food & Mood Centre, School of Medicine, Barwon Health, Geelong, Australia; 22 Orygen, Parkville, VIC Australia; 23 Centre for Youth Mental Health, University of Melbourne, Melbourne, VIC Australia; 24 School of Psychology, University of Birmingham, Birmingham, UK; 25 Department of General Practice and Primary Care, MDHS, University of Melbourne, Melbourne, VIC Australia; 26 MAGNET – Mental Health Australia General Clinical Trials Network, Deakin, Melbourne, VIC Australia; 27 Mental Illness Fellowship of Australia, Brisbane, QLD Australia; 28 Centre for Mental Health, Swinburne University of Technology, Melbourne, VIC Australia

**Keywords:** Master protocols, Effectiveness-implementation hybrid, Research methodology, Pragmatic trials, External validity, Trials within cohorts, TWiCs, Platform trials, Co-design, Dissemination, Translational research, Research network, Trial network, Research partnership, Research capacity building

## Abstract

**Background:**

Master protocols leverage a common trial infrastructure for launching multiple sub-studies. Translational research aims to progress scientific discoveries toward public health impact, which depends on establishing an intervention’s efficacy, effectiveness in real-world conditions, and successful strategies for implementation. While master protocols have been designed to improve the efficiency of clinical trials as sub-studies addressing a particular disease, their application with effectiveness-implementation hybrid studies is yet to be explored. The aim of this study was to develop recommendations for adapting mater protocol methods for effectiveness-implementation research.

**Methods:**

A method of consultation with translational research networks was undertaken between January and December 2024. Consideration was given to the requirements for service providers to engage in translational research, and how master protocols could support effectiveness-implementation hybrid sub-studies. The underlying rationale for potential adaptations is provided with reference to implementation frameworks, discussion of advantages and disadvantages, and summary recommendations.

**Results:**

Recommendations are proposed on establishing common trial infrastructure, aims and hypotheses, data collection, control groups, adaptive elements, and eligibility criteria. By leveraging cross-sectoral partnerships, co-producing research and dissemination, and incorporating adaptive elements, master protocols may offer a promising approach for accelerating progress along the translational research pipeline.

**Conclusions:**

The adaptation of master protocols for hybrid sub-studies could enable evidence-based interventions to be more effectively implemented in routine care settings. The feasibility of master protocols for effectiveness-implementation research is yet to be tested, and further development in this area is needed to trial the proposed methodology.

**Supplementary Information:**

The online version contains supplementary material available at 10.1186/s12874-025-02684-1.

## Introduction

**Fig. 1:**
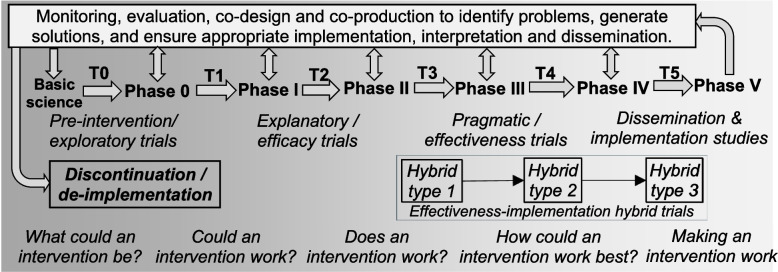
Translational research continuum or pipeline, with translational blocks (T0-T5) representing challenges needed to progress through different trial phases [2]. The term ‘intervention’ is used here to refer to the thing that is being trialled, including programs, practices, policies, products, principles, pills or procedures (7Ps) [7]. While some interventions may begin at basic science and follow this pipeline linearly (e.g. pills), others may begin at later phases and undergo cyclical improvement (e.g., programs, policies) or be discontinued. The placement of hybrid studies along this continuum is informed by Fortney et al [8], and the general research questions are informed by Brown et al. [7]. Descriptions of trial phases and translational blocks are tabulated in Additional file 1

Translational research broadly aims to progress discoveries and innovations toward public health impact [1]. While definitions of translational research have evolved over time [2], this process can be conceptualised as occurring along a ‘continuum’ or ‘pipeline’, with major challenges or ‘blocks’ needing to be overcome to progress between different stages (Fig. 1) [1]. Translational research aims to bridge basic science and exploratory first-in-human studies (T0), exploratory studies and clinical efficacy of interventions (T1), clinical efficacy and clinical effectiveness (T2), and clinical effectiveness and healthcare delivery (T3) [3], and healthcare delivery and broad scale adoption (T4) [2]. These blocks broadly align with clinical trial phases ranging from first-in-human to post-marketing and surveillance studies (Phase 0 to Phase IV). Trials in which individual monitoring is no longer required have been referred to as Phase V trials [4], and further barriers to adequate implementation of evidence-based recommendations must be continually addressed (T5). Importantly, this pathway is not linear, with some discoveries being adopted without late phase trials, and innovations generally beginning at higher phases or blocks (e.g., staff training intervention). ‘Backwards translation’ – in which knowledge produced at a later stage is ‘fed back’ to earlier stages – can inform research that is more relevant to policy, practice and public priorities [5]. Co-design and co-production are acknowledged as essential in this process [6].

A diverse range of research methods can be used to generate evidence relevant to traversing these blocks [7]. RCTs with individual randomisation have high internal validity for establishing efficacy when conducted under ideal conditions, and are also termed ‘explanatory trials’ [9]. When conducted in more realistic conditions, such as in routine care settings, RCTs have higher external validity for establishing effectiveness and are also termed ‘pragmatic trials’. RCTs can be made more pragmatic using a variety of design features, such as relaxing eligibility criteria or using cluster randomisation which increases contextual dependence. Trial designs can be characterised along an explanatory-pragmatic continuum (e.g. using PRECIS-Provider Strategies tool [10]), and higher pragmatism in trial design increases relevance to T3-T4 blocks. Once effectiveness is established, ‘implementation trials’ focus on testing implementation strategies designed to promote the use of evidence-based practices in routine care (T4-T5 blocks) [8]. To facilitate progression along this pipeline, trials can have a dual focus on clinical effectiveness and implementation [11]. Termed ‘effectiveness-implementation hybrid trials’, three types have been proposed: type 1 is primarily for testing clinical effectiveness while gathering data on implementation; type 2 incorporates co-primary aims of effectiveness and implementation strategies; and type 3 focuses on testing an implementation strategy while observing the impact of the intervention [12]. These different study types are identifiable by their primary outcomes, attention to fidelity, and use of implementation strategies (artificial versus practical; evidence-based versus novel) [8].

A universal challenge with high-quality and rigorous studies of any design is that they are resource intensive. Traditional parallel arm RCTs can be slow and inefficient because of their focus on a singular intervention, and studies with higher external validity such as cluster RCTs can be particularly complex undertakings. To improve research efficiency of explanatory trials, contemporary developments have led to ‘master protocols’ designed with multiple sub-studies targeting one or more disease sub-types within a well-defined population group [13]. Trial designs that use master protocols include ‘basket’, ‘umbrella’ and ‘platform’ trials [11, 14], with platform trials incorporating within-trial adaptations based on interim analyses, or addition of new treatment arms over the course of the trial (‘open’ or ‘perpetual’ design). Compared with traditional RCTs, master protocols may improve trial efficiency by sharing operational elements (i.e. ‘common trial infrastructure’), with some designs also incorporating a shared control group across sub-studies (i.e., ‘common control group’, particularly in umbrella trials) [13]. Master protocols have been commonly used for investigating experimental drugs in oncology using phase II or III trial designs (relevant to T2-T3 blocks) [15] and recently in infectious diseases [16]. However, their potential to streamline effectiveness-implementation hybrid studies and accelerate evidence generation relevant for T3-T5 blocks remains largely unexplored. Therefore, the aim of this study was to propose adaptations for utilising master protocols for effectiveness-implementation hybrid studies.

## Methods

As this was an exploratory study designed to propose adaptation of innovative trial methods, consultation with expert networks was undertaken to develop recommendations for adapting master protocol methods for hybrid studies. Consultation was conducted in groups in-person and online, involving: (i) discussion about master protocols and implementation methods, (ii) consideration of service provider requirements to effectively support translational research, (iii) building research capacity, and (iv) dissemination mechanisms. The lead author (JC) took notes from the consultations and engaged in critical discussion with the authorship group who have experience across master protocols, implementation science and statistics; however, consultations were not recorded, and no qualitative analysis was conducted. Reporting in this manuscript is guided by the Standards for Reporting Qualitative Research (Additional file 2).

Consultation was undertaken between January and December 2024 with the Co-design and Research Translation Alliance in Mental Health (CoRTA-MH), Implementation and Translation Network within the ALIVE National Centre for Mental Health Research Translation (ALIVE-ITN), and the Equally Well Global Leadership Exchange group (EW-GLE). Membership of these committees consisted of health service staff, researchers with broad expertise in health service sectors, and people with lived experience. A total of 22 consultees completed a questionnaire on professional demographics (Additional file 3) and provided consent for individual acknowledgement: most had research/academic backgrounds (n = 9), followed by mental health professionals (Allied Health or Medical; n = 7) and organisational or systems change advocates (n = 6). Data on individuals who engaged with the consultation but did not complete the questionnaire are unavailable. Ethical approval was provided by the Griffith University Human Research Ethics Committee (2024/160).

Findings are presented in seven sections: (1) conceptual overview; (2) establishing common trial infrastructure; (3) framing aims and hypotheses; (4) embedding data collection; (5) forming control groups; (6) incorporating adaptive elements; and (7) eligibility criteria. Where it was considered to improve clarity, the rationale is grounded in constructed examples of hybrid sub-studies (Table 1). Severe mental illness (SMI) was chosen for these examples because of the authorship group’s expertise; however, the examples could apply for any other health condition.

**Table 1:** Constructed example

This constructed example of a master protocol involves partner organisations across hospital and health services and community-managed organisations. The translational research focus is on psychosocial interventions for people with severe mental illness (SMI), including schizophrenia, bipolar disorder, major depression. Three hybrid type sub-studies will be considered:
***Hybrid type 1****:* Exercise intervention for people with SMI, implemented using exercise physiologists employed within the study * Primary aim*: To evaluate the effectiveness of the intervention for improving quality of life of recipients * Hypothesis:* Exercise improves quality of life, mediated by psychological distress
***Hybrid type 2****:* Physical activity coaching intervention for people with SMI, with two different implementation strategies: ‘high intensity’ involving online training for existing staff *plus* persuasive communication; ‘low intensity’ involving online training only * Co-primary aims*: To: (i) evaluate the effectiveness of the intervention for improving quality of life of recipients; (ii) evaluate the implementation strategy for improving fidelity of the intervention * Hypotheses:* (i) physical activity improves quality of life; (ii) the ‘high intensity’ strategy is more effective at improving fidelity, and the perceived implementability mediates the relationship between implementation strategy and fidelity
***Hybrid type 3****:* Diet education intervention for people with SMI, with two different implementation strategies: ‘high intensity’ involving online training for existing staff *plus* financial incentives; ‘low intensity’ involving online training only * Primary aim*: To evaluate the implementation strategy for improving adoption of the intervention * Hypothesis:* The ‘high intensity’ strategy is more effective at improving adoption, and implementation climate moderates the relationship between implementation strategy and adoption

## Results

### Conceptual overview

Traditional RCTs have been described as ‘intervention-focused’, with the generalised research question “*can this intervention offer benefit over current standard of care or placebo?*” [17]. Master protocols expand this scope by enabling investigation of multiple sub-studies in one or more disease sub-types. In this way, master protocols have been described as ‘disease-focused’, with the generalised research question “*what is the best intervention for a given disease?*’’ [17]. Analogously, hybrid studies may be described as ‘innovation-focused’ to include consideration of contextual factors related to implementation. A master protocol for hybrid studies could expand this scope to become ‘service-focused’, enabling investigation of multiple hybrid sub-studies with one or more service types designed for a specific population group (which may include multiple sub-groups; Table 1). The generalised research question would then be “*what interventions can be effectively implemented across different service types to improve outcomes for recipients?*”. This approach encompasses cross-sectoral strategies to improve outcomes for target populations, potentially including different healthcare settings and/or health, human and social service types.

### Establishing common trial infrastructure

A common trial infrastructure comprises shared processes and resources for multiple trials, including protocol development, approvals and agreements, recruitment, data collection and management. Service providers are essential because of their direct connection with communities and pathways for translation. Hybrid sub-studies may require considerable commitment from service providers, particularly for type 2 and type 3 studies focusing on implementation. Endorsement from management and direct staff involvement may be needed, which may also include participating in surveys or focus groups to evaluate implementation outcomes. Establishing service providers as *partner organisations* using an overarching agreement could embed collaborative relationships and improve stability of the common trial infrastructure.

Partner organisation relationships are built over time and are supported when there is mutual benefit. Mutual benefit can be realised in several ways, including co-production, research capacity building, and dissemination activities. Co-producing the research with staff and recipients of their services ensures that the research aligns with the priorities of partner organisations and their recipients. Research capacity building can improve research engagement and evidence-based practice, and enhance ongoing partner organisation involvement [18]. Dissemination activities enhance value for partner organisations by identifying areas of unmet need, monitoring recipient outcomes, and informing quality improvement and policy translation. A master protocol could outline ongoing co-production of sub-studies, capacity building, and dissemination activities.

Hybrid sub-studies may include trials of complex service model approaches, such as collaborative care, care coordination, or stepped-care models. The complexity of interventions that may be trialled in sub-studies necessitates a more complex trial infrastructure, which may require multiple partner organisations across different settings. Diverse partnerships may delay research approvals and governance arrangements because of the number of organisational signatories and operational differences. However, once established, a master protocol could play a critical role in facilitating translational research to improve system integration and continuity of care. Master protocols streamline the addition of sub-studies via amendments to ethics committees and contract variations to governance agreements, potentially offering improvements to the efficiency of such trials.

#### Recommendations

- Common trial infrastructure should involve partner organisations across different service settings, enabling evaluation of cross-sectoral service models to address the needs of the population group outlined in the master protocol.
- Partnership should be strengthened by formalising commitment toward a common vision of enabling translational research, underpinned by the master protocol with collaborative governance arrangements.
- The master protocol should be co-produced with partner organisations and recipients of their services, and stipulate processes such as co-production of sub-studies, research capacity building, and research dissemination activities.

#### Considerations

- Collaborative relationships take time to establish, particularly if partners are across sectors or service settings; involving peak or commissioning bodies may facilitate collaborations.
- Co-production, research capacity building, and dissemination activities require resourcing that may not be included in conventional trial funding schemes; however, some funding opportunities may specifically support these activities.
- A consortium arrangement could be used to formalise partnerships, and engagement could be enhanced by additional branding, governance and communication strategies.

### Framing aims and hypotheses

**Fig. 2:**
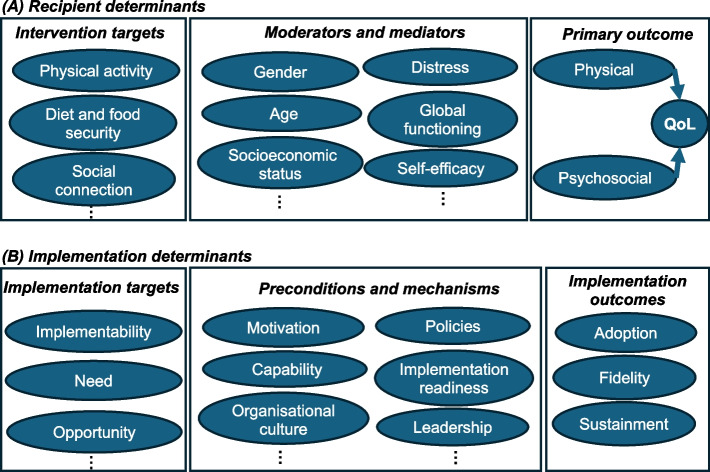
Example determinant models that could be developed based on co-designed priorities and existing evidence and theory. These simplified examples are broadly informed by the Consolidated Framework for Implementation Research [20] and causal pathway models from Lewis et al [19]. **A*** Recipient determinants* may be biopsychosocial and behavioural factors that are hypothesised to be related to the intervention targets (co-designed priorities) and primary outcome (QoL: quality of life), which could be addressed through a variety of interventions. **B*** Implementation determinants* may be individual, inner setting and outer setting constructs that are hypothesised to be related to the implementation targets and implementation outcomes, which could be addressed through a variety of implementation strategies

Hybrid sub-studies may aim to improve effectiveness outcomes (e.g. quality of life) and/or implementation outcomes (e.g. adoption) by influencing their associated determinants. Because of the potential diversity of sub-studies, constraints need to be established in the master protocol to improve coherence between the overarching aim of the master protocol and the sub-study aims. Multi-level determinant models could be used to constrain the design features and operational elements by establishing parameters for the measurable mediators, moderators, preconditions, and outcomes (proximal versus distal) [19]. These determinant models would be developed for each master protocol and stipulated a priori, tailored to the: (i) recipient group, (ii) partner organisations involved, (iii) sub-studies intended, (iv) data collection feasibility, and (v) the selected implementation frameworks and health-related theories. For illustration purposes, simplified examples are provided in Fig. 2.

Framed by the determinant models, aims and hypotheses could be hierarchically ordered in the master protocol. An *overarching aim* of the master protocol may be to evaluate these determinant models using multi-level modelling, structural equation modelling, pathway modelling or mixed methods such as matrixed multiple case study (MMCS) approach [21]. *Secondary-level aims* would be to validate theoretical relationships between the determinants, and *tertiary-level aims* would be to understand the influences on these determinants for each sub-study. Because hybrid sub-studies may focus on effectiveness and/or implementation outcomes, aims may be underpinned by recipient-level, deliverer-level or cluster-level hypotheses related to the determinants being considered. Some examples are provided in Table 2.

**Table 2:** Example aims and hypotheses that could be defined in a master protocol for hybrid sub-studies

	**Research questions**	**Aims**	**Objectives**	**Example hypotheses**		
				***Overarching hypothesis***		
*Overarching aim*	How well do the determinant models predict the outcomes?	To evaluate the multilevel determinant models	To identify proximal and distal influences on the outcomes	The determinant models are reasonable representations of the outcomes of interest		
				*Recipient-level*	*Deliverer-level*	*Cluster-level*
*Secondary-level aims*	What are the inter-relationships between key determinants?	To evaluate the relationship between determinants	To identify proximal and distal influences on the determinant of interest, and moderators, mediators for these relationships	Exercise improves quality of life, mediated by psychological distress	Perceived implementability the relationship between implementation strategy and fidelity	Implementation climate moderates the relationship between implementation strategy and adoption
*Tertiary-level aims*	*Hybrid type 1 and 2*^*a*^: What intervention characteristics are effective when implemented in routine care settings, and for whom?	To evaluate the intervention effectiveness	To understand the mechanisms through which recipient/intervention determinants influence recipient/intervention outcomes	Perceived acceptability of the intervention improve attendance	-	-
	*Hybrid type 2*^a^* and 3:* What implementation strategies are effective at improving implementation outcomes, and in what context?	To evaluate implementation strategies	To understand the mechanisms through which implementation determinants influence implementation outcomes	-	Persuasive communication improves perceived implementability	Staff turn-over negatively impacts implementation climate

‘Recipient-level’ refers to the intended beneficiaries of interventions, referred to in different contexts as consumers, clients, patients, participants etc.; ‘deliverer-level’ refers to staff, specifically those who are the target of implementation strategies designed to support delivery of interventions; ‘cluster-level’ refers to group-level units (e.g. teams or organisations) and outcomes that are dependent on collective processes (e.g. average attitudes or capacity of the group). Hybrid type 1 studies only have recipient-level hypotheses because they aim to evaluate clinical effectiveness while collecting implementation data; hybrid type 2 studies can have recipient-, deliverer- and/or cluster-level hypotheses because of co-primary effectiveness and implementation aims; hybrid type 3 studies only have deliverer- or cluster- level hypotheses because of their primary focus on implementation outcomes. Figure 2 provides general diagrammatic representation of determinants relevant to the example hypotheses

^a^Hybrid type 2 studies involve co-primary aims; hence hypotheses have been combined with hybrid type 1 or 3 to reduce redundancy

#### Recommendations

- Aims should be hierarchically ordered in the master protocol, with the overarching aim and secondary-level aims framed by a priori multi-level determinant models related to intervention outcomes and implementation outcomes.
- Multi-level determinant models should be theory-informed and designed for each master protocol, considering the common trial infrastructure and target population group.
- Tertiary-level aims should be specific to each sub-study, and require deeper investigation to better understand relationships between the outcomes and relevant determinants.

#### Considerations

- Developing the determinant models may require considerable investigation and planning, but would improve clarity for partner organisations and set parameters for data collection and sub-studies.
- Constraining sub-studies to those that address the determinants improve coherence with the overarching aim, but also reduces flexibility and responsiveness to support a diverse range of sub-studies.
- The multi-level determinant models may include many interactions, requiring a substantial amount of high-quality data to evaluate with adequate rigor, thereby reducing feasibility of addressing the overarching aim.

### Embedding data collection

**Fig. 3:**
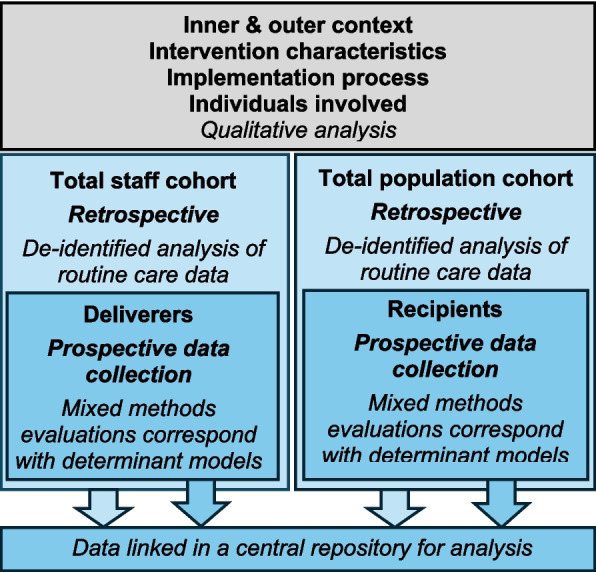
Schematic of potential data sources and collection methods related to staff, recipients, and the organisational context; domains informed by the Consolidated Framework for Implementation Research [20]

Hybrid sub-studies require multiple sources of data collection to evaluate effectiveness and implementation outcomes. To address the aims, the research measures would correspond with the determinants and could involve a range of qualitative and quantitative, prospective and retrospective evaluations (Fig. 3). Data would be collected longitudinally to measure changes in the determinants over time, which could be used to address the overarching aim and secondary-level aims stipulated in the master protocol. These data could also be used to address tertiary-level aims provided that sub-studies only target these determinants as potential mechanisms for improving the outcomes. Additional measures could be specified for each stub-study depending on tertiary-level hypotheses. While much of these data would be collected specifically for research, establishing a Clinical Quality Registry [22] for dual research and routine care purposes could improve applicability to practice and support embedded quality improvement models such as learning health systems [23].

Each data source would involve different ethical and governance considerations. Qualitative analysis of contextual factors, organisational policy, implementation process and intervention characteristics may not require formal approval, whereas measurement of attitudes or behaviours would require individual consent. Routine care data related to staff or recipients may be accessible for retrospective analysis with waivers of consent, and linking with prospectively collected data may be possible with individual consent. Data would be collected longitudinally with either the same individuals or clusters depending on the outcome; for example, changes in quality of life could be measured longitudinally with recipients, whereas organisational culture may be measured longitudinally with the teams (i.e. clusters) which may have different individuals at each timepoint. Data linkage in a central repository established for the master protocol would allow data pooling to address aims and hypotheses.

#### Recommendations

- Research measures should correspond with determinants outlined in the multi-level determinant models, and data should be collected longitudinally over the course of the master protocol.
- Hybrid sub-studies should include additional measures and evaluation related to the intervention to address tertiary-level aims.
- A central database should be established for data linking across sub-studies to address the overarching aim and secondary-level aims.

#### Considerations

- Establishing a registry that serves dual purposes for research and routine care could improve feasibility of data collection and inform quality improvement; however, challenges related to data security across organisations would need to be overcome.
- If the common trial infrastructure includes cross-sectoral partnerships, contextual differences across settings could be characterised and their impact on implementation outcomes evaluated, thereby facilitating context-specific implementation recommendations.

### Forming control groups

Hybrid sub-studies span the effectiveness-implementation continuum, and control groups may be at the individual or cluster level depending on the hypotheses being tested. Allocation to intervention or control arms may be randomised (experimental) or non-randomised (quasi-experimental) depending on the design chosen for each hybrid sub-study [12]. Allocation of individuals or clusters for each sub-study would be timed with data collection to ensure baseline data is collected prior to implementation. Partner organisations across different service settings may involve considerable variation in routine care processes, which could be quantified by examining longitudinal data and used to inform stratification to ensure balance. Routine care data may be analysed retrospectively as ‘historical controls’ if data quality is suitable.

**Fig. 4:**
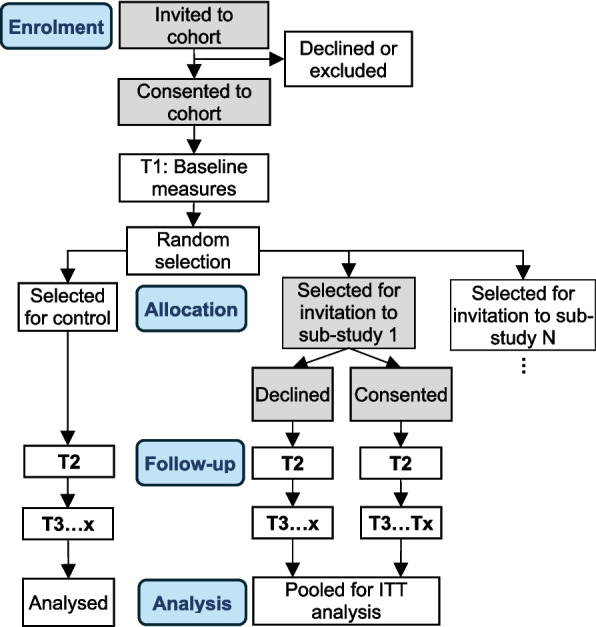
Participant flow diagram for a *trials within cohorts* design. The cohort study may have up to x longitudinal assessments (T1 to Tx), with cohort participants being randomly selected to be invited to one of N sub-studies after baseline assessments. Note the sub-study requires a second stage of consent

Hybrid type 1 sub-studies investigate effectiveness outcomes while exploring implementability. While quasi-experimental methods could be used, randomised designs would be highly feasible because of the common trial infrastructure and embedded data collection. Given that the master protocol involves longitudinal data collection with recipients, a random selection of the cohort could be invited to sub-studies, with those not randomly selected analysed as the control group. This is called a ‘*trials within cohort*s’ (TWiCs) design, and requires a two-stage consent process and data linking between the longitudinal study and sub-study [24], which is an ethical approach to conducting pragmatic trials [25]. TWiCs designs have several pragmatic advantages compared with conventional RCTs, and more closely parallel routine care in which recipients are offered an intervention rather than randomly allocated. Recruitment bias may be minimised because the decision to participate in the longitudinal study would not be influenced by the nature of the intervention, which can be important for highly desirable interventions. A common control group may also be formed using this design, allowing more participants to be selected for one of the available sub-studies. Consider the hybrid type 1 exercise intervention (Table 1): if people with SMI are initially recruited to a longitudinal cohort study, the decision to participate would be dependent on the perceived benefit or burden of the research (e.g., assessments, data security, remuneration, etc.). If a random selection of the cohort is then invited to the exercise sub-study, the participant is afforded complete autonomy to engage in the intervention without the prospect of being excluded via random allocation, and the experience of other cohort participants (controls) remains unchanged. An example CONSORT-style participant flow diagram for a TWiCs study is shown in Fig. 4.

Hybrid type 2 sub-studies involve co-primary effectiveness and implementation outcomes, allowing for the simultaneous testing or piloting of implementation strategies within an effectiveness trial. Because co-primary outcomes are at the recipient level (for effectiveness) and cluster level (for implementation), randomised and/or quasi-experimental designs may be considered for each level separately. Two levels of randomisation within the same hybrid type 2 study may improve robustness for both co-primary aims, but would substantially increase complexity and potential for contamination of the recipient-level control group. It is likely that the only situation in which two levels of randomisation would be considered is when the cluster-level intervention can be isolated from recipient-level controls. Consider the hybrid type 2 physical activity coaching intervention (Table 1): if teams (clusters) are randomised to the implementation strategy (‘high’ or ‘low’ intensity staff training) and recipients within each cluster are also randomised to control or intervention groups, control recipients could be isolated from the cluster-level intervention by ensuring that only trained staff (deliverers) provide services for recipients randomised to the intervention arm.

Hybrid type 3 sub-studies primarily investigate implementation outcomes while also collecting effectiveness data. Cluster randomised or group-level quasi-experimental designs may be chosen to test the associated deliverer-level or cluster-level hypotheses. Mutual benefit for partner organisations may be an important consideration; therefore, ‘active’ control conditions in which different implementation strategies are trialled may be favourable over ‘routine care’ controls to ensure all clusters receive the intervention. Stepped-wedge cluster randomised trials could also be a suitable alternative. Quasi-experimental designs (e.g., interrupted time series or non-equivalent control group) are still invaluable for informing implementation recommendations, and may be considered if resource limitations or other constraints reduce feasibility of an adequately powered experimental design. Consider the hybrid type 3 diet education intervention (Table 1): random allocation of sites may be stratified by service type (e.g., community vs hospital service) and contextual determinants (e.g. high vs low implementation readiness), then different implementation strategies used for ‘intervention’ and ‘active control’ arms (high vs low intensity).

#### Recommendations

- Sub-studies should be coordinated with longitudinal data collection to ensure appropriate baseline data has been collected prior to launching new sub-studies.
- Hybrid type 1 sub-studies should employ TWiCs methodology because of pragmatic methodological benefits, potentially allowing the formation of a common control group.
- Hybrid type 2 sub-studies should define control groups at the recipient and cluster levels to address the hypotheses, minimising potential contamination of the recipient control group caused by group-level implementation.
- Hybrid type 3 sub-studies should be cluster randomised parallel-group or stepped wedge trials, unless logistical or resource constraints reduce feasibility of using a randomised design.

#### Considerations

- Historical control groups formed using routine care data could be used where appropriate,.
- Mutual benefit for partner organisations should be considered in trial design, and ‘active’ rather than ‘routine care’ control conditions may be appropriate for hybrid type 2 or 3 trials.
- Two levels of randomisation may be considered for hybrid type 2 studies investigating both effectiveness and implementation outcomes; however, this may only be for specific designs in which the cluster-level intervention can be isolated from the recipient-level control group.

### Incorporating adaptive elements

Adaptive features can support trial agility by leveraging results that accumulate at pre-specified interim time-points and are evaluated in accordance with pre-specified rules. An adaptive feature characteristic of platform trials is the addition of treatment arms over the course of the master protocol. The maximum number of concurrent interventions may be stipulated in the master protocol, considering feasibility of recruitment, sample size requirements, resourcing available, and maintaining integrity of analyses. The addition of sub-studies is dependent on subsequent funding and resourcing, and for hybrid master protocols, would be guided by the overarching determinant model. Because hybrid type 2 and 3 sub-studies have a greater dependency on the organisational context, an additional consideration is maintaining clarity for partner organisations and reducing potential confusion caused by multiple concurrent trials.

Multiple hybrid type 1 sub-studies could be added relatively simply: longitudinal cohort participants would be randomly selected for invitation to one of the type 1 sub-studies available (Fig. 4). Implementing a hybrid type 1 sub-study concurrently with type 2 or 3 sub-studies may involve both individual and cluster level randomisation, increasing trial complexity. Consider the example in which hybrid type 1 and 3 sub-studies are operating concurrently (Table 1): the cluster randomised type 3 study involves staff training in diet education interventions, and the type 1 study involves an exercise intervention offered directly to randomly selected recipients. The recipient control group may be exposed to a staff-delivered diet education intervention, which may impact the primary outcome through similar mechanisms as the exercise intervention (e.g. improved self-efficacy). This could reduce the observed effect of the exercise intervention, increasing the potential for type 2 error. However, this may be partially mitigated by adjusting for these changes in analyses, i.e., diet quality and self-efficacy would be measured longitudinally guided by the determinant model, and changes to routine care may be characterised using routine care data. The potential impact on methodological integrity would need to be considered for the addition of each sub-study.

Adaptive features unique to hybrid sub-studies may include adaptive implementation. A range of adaptive implementation strategies have been explored [26], and may involve graduating an ineffective strategy to a new implementation strategy informed by interim evaluation demonstrating poor adoption, fidelity, sustainability etc. For example, previous studies incorporating adaptive implementation used random allocation of non-responsive sites to a more resource intensive strategy approach [27–29]. If the common trial infrastructure includes partner organisations across different regions or settings, ‘adaptive scaling’ could involve a type 3 sub-study progressing from one region or setting type to another. While there are no known studies published incorporating adaptive scaling, it could be informed by interim analyses defining implementation success, then applying the same strategy in another setting to evaluate differences. Adaptative features may be specific to each sub-study rather than pre-specified in the master protocol thereby applying to all sub-studies.

Platform trials have incorporated a range of other adaptive elements that may be useful for translational research. For example, updating target sample sizes [30], terminating apparently ineffective treatments, updating allocation ratios to prioritise more promising treatments or participant cohorts that benefit most, or ‘dose escalation adaptivity’ where medication dose is increased according to pre-specified criteria. These adaptive strategies may have applicability for some hybrid sub-studies, particularly hybrid type 1, which is open to exploration.

#### Recommendations

- The addition of new sub-studies should be included as an adaptive element, with restrictions on the number of concurrent sub-studies dependent on feasibility (e.g. recruitment potential) and clarity for partner organisations.
- Adding new sub-studies should be dependent on alignment with the overarching determinant model and maintaining integrity of analyses in the context of concurrent sub-studies.

#### Considerations

- Careful consideration about potential interference across sub-studies would be needed to maintain methodological integrity, and be managed by measuring the relevant determinants for adjusting in analyses, and separating trial sites or timings to minimise interference.
- Many adaptive elements commonly utilised in platform trials may have applicability to hybrid type 1 sub-studies, and hybrid type 2 and 3 sub-studies may benefit from adaptive implementation.

### Eligibility criteria

Eligibility criteria must accommodate the aims of hybrid type 1, 2 or 3 sub-studies. The most pragmatic eligibility criteria would include anyone who would likely receive the intervention if it was being offered in routine care. Eligibility criteria for longitudinal data collection could therefore simply be that participants must be accessing services that comprise the common trial infrastructure. This aligns with the proposed ‘service-focused’ scope, and improves external validity which is important for addressing the aims of hybrid type 2 and 3 sub-studies.

**Fig. 5:**
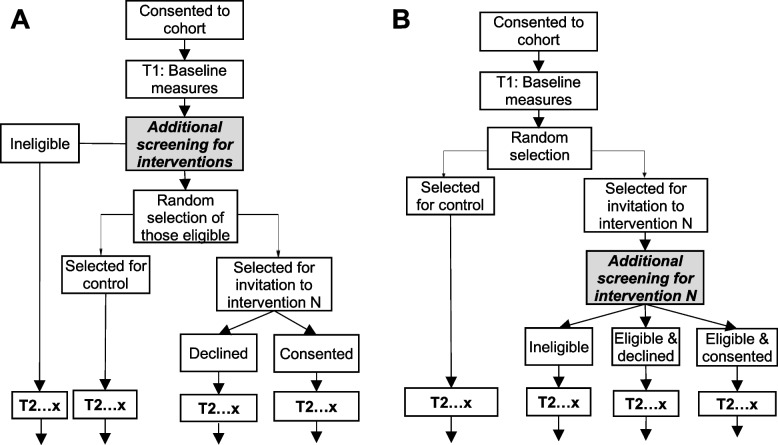
Participant flow diagram for a *trials within cohorts* design being used for hybrid type 1 sub-studies, with second stage consent for the sub-studies placed either: (**A**) prior to random selection, or (**B**) after random selection. The longitudinal cohort includes x assessments (T1 to Tx), and participants are randomly selected for one of N potentially concurrent sub-studies

Additional eligibility criteria may need to be imposed for some sub-studies, particularly for type 1 sub-studies which are less pragmatic by design. For example, participants with medical contraindications may be excluded from an exercise sub-study but not the longitudinal study. This would require a second stage of eligibility screening for sub-studies, which could be applied prior to random selection or after randomisation (Fig. 5). It is important to note that participants ineligible for any available sub-studies would still be included in analyses addressing the overarching aim of the master protocol. The advantages and disadvantages of each of these approaches are summarised in Table 3.

**Table 3:** Comparison of second stage eligibility criteria applied before or after randomisation within a TWiCs design

*Screening prior to randomisation*
*Advantages* • Recruitment to the sub-study is efficient because only participants eligible for each intervention are randomly selected for the intervention or control condition • The feasibility of trials with highly selective groups (e.g. rare diseases) may be improved because all participants in the longitudinal cohort are screened *Disadvantages* • Burden is increased because screening measures for all concurrent sub-studies must be administered with all participants in the longitudinal cohort, but participants may only be randomly selected for one intervention • Additional screening measures may need to be added to the longitudinal study as new sub-studies are added • Because random selection is dependent on meeting screening criteria, a common control group may be prone to bias (via altered prevalence of the screening risk factors); each sub-study may therefore need a separate control group
***Screening after randomisation***
*Advantages* • Feasible if the proportion of ineligible participants is anticipated to be low • Burden is minimised by only administering relevant screening measures with participants randomly selected for an intervention • The control group remains representative of the sample recruited, and could be used as a common control group *Disadvantages* • Prevalence of the screening risk factors would be unknown for the control group because only those selected for the intervention undergo additional screening, which may introduce an unacceptable level of bias depending on the screening criteria • Trial efficiency may be reduced because a proportion of participants selected for the intervention condition would be ineligible

*TWiCs* Trials within cohorts

#### Recommendations

- Eligibility criteria for the longitudinal cohort should be broad, aiming to recruit a sample representative of the population group accessing services that form the common trial infrastructure.
- Additional screening for sub-studies should be minimised, but if necessary should be:
  - applied after randomisation if: (i) few participants are expected to be ineligible, (ii) all participants allocated to the intervention are pooled for intention-to-treat analysis, and (iii) measuring the prevalence of the screening factor/s is not important for the control group.
  - applied before randomisation if the criteria are moderate-highly selective, with the control group maintained separately for that sub-study.

#### Considerations

- Applying additional screening after randomisation may improve generalisability if the process reflects routine care; for example, if medical screening is routinely conducted after referral to an exercise physiologist, results may be more generalisable to recipients referred to a service if sub-groups are pooled for analysis. Further sub-group analyses with those who participated (e.g. per protocol analysis) could be conducted.
- Employing both screening options in different concurrent sub-studies may be possible but would introduce additional complexities needing two randomisation steps in the participant flow.

## Discussion

This article explored how master protocols could be adapted to support translational research along the effectiveness-implementation continuum. Embedding implementation evaluation into ‘conventional’ master protocols would also be beneficial, and indeed there are calls to improve implementation evaluation in all clinical trials [31]; however, existing master protocols do not focus on effectiveness-implementation hybrid studies for generating evidence relevant for T3-T5 blocks. Despite the challenges involved with master protocols, such as complexity in planning, funding, and reporting, master protocols adapted for hybrid sub-studies could improve translation of evidence into practice. Critical considerations toward this goal were provided for establishing common trial infrastructure; framing aims and hypotheses; embedding data collection; forming control groups; incorporating adaptive elements; and eligibility criteria.

To the authors’ knowledge, master protocols have not been specifically adapted for hybrid studies; however, some studies have included similar features described in this manuscript. For example, TWiCs designs involve a longitudinal cohort as the ‘platform’ from which to launch multiple RCTs; however, TWiCs have not specifically sought to be service-focused by evaluating implementation determinants and enabling hybrid type 1–3 trials, including cluster randomised designs. The STIMULATE-ICP master protocol study involved two randomisation levels including a cluster randomised pragmatic trial of an integrated care pathway with a nested adaptive platform randomised drug trial [32]: random allocation was to four different conditions at the cluster level and four different drug treatments at the individual level. While this study included effectiveness research, it did not extend into implementation research which would require more comprehensive evaluation of implementation determinants, and it is not clear if this master protocol could support multiple pragmatic trials. We propose that master protocols for hybrid sub-studies should seek to embed data collection into routine practice with service providers to enable research along the translational continuum, guided by an overarching aim and multi-level determinant models.

Ultimately, the adaptation of master protocols for hybrid sub-studies (perhaps ‘hybrid master protocols’) could enable evidence-based interventions to be more effectively implemented in routine care settings. Hybrid master protocols may be complementary to Learning Health Systems which aim to embed the use of service data and patient engagement for continual quality improvement [23]. A limitation of this work is that hybrid master protocols are yet to be feasibility tested; however, this article provides critical considerations toward that goal, which the authorship group are exploring through a city-wide translational research consortium called the *Co-design and Research Translation Alliance in Mental Health* (CoRTA-MH). Feasibility testing of hybrid master protocols could focus on characterising trial efficiency informed by an established conceptual framework [33].

The recommendations presented were based on consultation with expert research networks and learnings from collaboration with partner organisations in mental health service delivery. Consultations were not recorded, and no qualitative analyses were conducted to arrive at the recommendations and considerations. The authors do not purport to have considered all potential applications of hybrid master protocols, but rather, intend for this manuscript to stimulate further debate and commentary about developing translational research architecture for impacting policy and practice.

## Conclusion

By leveraging a common trial infrastructure founded on cross-sectoral partnerships, co-designing and co-producing research and dissemination pathways, embedding data collection and incorporating adaptive elements for trial agility, master protocols may prove critical to addressing translational challenges across a range of health, human and social service settings. The feasibility of master protocols for hybrid studies is yet to be trialled, and the recommendations provided in this manuscript could guide this next step.

## Supplementary Information

Supplementary Material 1.

Supplementary Material 2.

Supplementary Material 3.

## Data Availability

Not applicable.

## Abbreviations

CFIR: Consolidated Framework for Implementation Research
MMCS: Matrixed multiple case study
QoL: Quality of life
RCT: Randomised controlled trial
SMI: Severe mental illness
TWiCs: Trials within cohorts
