# Supplementary material for: Could master protocols be adapted for effectiveness-implementation hybrid studies?
Source: BMC Med Res Methodol. 2025 Nov 18;25:258. doi: 10.1186/s12874-025-02684-1 (PMC12625322; doi:10.1186/s12874-025-02684-1)
Supplement: Supplementary file 1 — Supplementary Material 1. [file 12874_2025_2684_MOESM1_ESM.docx]

| **Table S1: Overview of clinical trial phases**[**^1^**](#_ENREF_1) **and translational research blocks** | | | |
| --- | --- | --- | --- |
|  | **Description** | **Objectives and challenges** | **“T” block challenges**[^2^](#_ENREF_2) |
| ***Basic science*** | Laboratory research involving in vitro studies, animal models or computational simulations. | - To advance fundamental knowledge that may lead to new technologies and treatments. | **T0 challenges**:   - Translating findings from animal models to humans - Replicating and validating results |
| ***Phase 0*** | Proof of concept or exploratory studies to gather preliminary data about a new drug or treatment using microdoses to minimize risks; small samples (n~10-20)[^3^](#_ENREF_3). | - Assess pharmacokinetics and pharmacodynamics. - Determine whether the drug reaches its intended target and modulates it as expected. |  |
|  |  |  | **T1 challenges**:   - Securing funding for early-stage research with uncertain clinical applicability - Navigating regulatory requirements |
| ***Phase 1*** | The initial stage of testing a new treatment in humans; small samples (n~20-80). Generally not RCT designs but may include randomisation if considered safe and ethical[^4^](#_ENREF_4). | - Evaluate the safety of the drug or treatment - Determine the safe dosage range - Identify potential side effects - Study how the drug is metabolized and processed in the body |  |
|  |  |  | **T2 challenges**:   - Recruiting diverse patient populations for trials - Addressing variability in patient outcomes - Translating trial results into practical clinical recommendations |
| ***Phase 2*** | The second stage of testing a new treatment in humans. May be randomised controlled trials, but typically do not use placebos, focusing instead on the active treatment. They involve a larger group of participants, usually ranging from 25 to 300 people[^5^](#_ENREF_5). | - To assess the efficacy of the treatment, determining if it works as intended. - To further evaluate the safety and side effects in a larger group. - To determine the optimal dose for its intended use. |  |
|  |  |  | **T3 challenges**:   - Overcoming resistance to change in healthcare settings - Addressing contextual factors that influence adoption - Developing effective dissemination strategies |
| ***Phase 3*** | Comparative efficacy studies designed to evaluate effectiveness and safety of compared to the current standard of care. Larger samples (n>300) and commonly using RCT designs. | - Confirm efficacy and effectiveness of the new treatment - Monitor side effects and safety in a larger population - Collect information for safe use of the intervention |  |
|  |  |  | **T4 challenges**:   - Designing and implementing large-scale population studies - Addressing health disparities and social determinants of health - Translating research findings into policy changes |
| ***Phase 4*** | Post-marketing surveillance studies often take place in healthcare settings when the treatment is publicly available; can be observational or RCT designs[^6^](#_ENREF_6). | - Monitor long-term safety and effectiveness in real-world settings - Identify rare or delayed adverse reactions - Evaluate the treatment's performance across diverse populations - Assess cost-effectiveness. |  |
|  |  |  | **T5 challenges**:   - Translating research policy into changes in routine practice and processes. - Being responsive to community needs, expectations and policy. - Reducing inequity and inequality. |
| ***Phase 5*** | Effectiveness and implementation research aiming to integrate the treatment into a large number of public health practices. [^7^](#_ENREF_7) | - Integrate a new clinical treatment into routine care. - Determine whether it can be successfully implemented in different settings. - Improve public health outcomes by translating research findings into widespread clinical practice. |  |

1. Kandi V, Vadakedath S. Clinical trials and clinical research: a comprehensive review. *Cureus*. 2023;**15**.

2. Fort DG, Herr TM, Shaw PL, Gutzman KE, Starren JB. Mapping the evolving definitions of translational research. *Journal of clinical and translational science*. 2017;**1**:60-6.

3. Kummar S, Rubinstein L, Kinders R*, et al.* Phase 0 clinical trials: conceptions and misconceptions. *The Cancer Journal*. 2008;**14**:133-7.

4. Iasonos A, O’Quigley J. Randomised Phase 1 clinical trials in oncology. *British Journal of Cancer*. 2021;**125**:920-6.

5. McShane L, Hunsberger S. An overview of Phase II clinical trial designs with biomarkers. *Design and Analysis of Clinical Trials for Predictive Medicine Chapman and Hall/CRC*. 2015:71-87.

6. Henry BM, Lippi G, Nasser A, Ostrowski P. Characteristics of Phase IV clinical trials in oncology: an analysis using the ClinicalTrials. gov registry data. *Current Oncology*. 2023;**30**:5932-45.

7. Rohilla A, Singh RK, Sharma D, Keshari R, Kushnoor A. Phases of clinical trials: a review. *International Journal of Pharmaceutical, Chemical & Biological Sciences*. 2013;**3**.
