# Supplementary material for: Could master protocols be adapted for effectiveness-implementation hybrid studies?
Source: BMC Med Res Methodol. 2025 Nov 18;25:258. doi: 10.1186/s12874-025-02684-1 (PMC12625322; doi:10.1186/s12874-025-02684-1)
Supplement: Supplementary file 2 — Supplementary Material 2. [file 12874_2025_2684_MOESM2_ESM.docx]

| **Standards for Reporting Qualitative Research (SRQR)*** | |
| --- | --- |
| **Title and abstract** | **Researcher response** |
| ***Title*** - Concise description of the nature and topic of the study Identifying the study as qualitative or indicating the approach (e.g., ethnography, grounded theory) or data collection methods (e.g., interview, focus group) is recommended | Title reflects the question of inquiry |
| ***Abstract*** - Summary of key elements of the study using the abstract format of the intended publication; typically includes background, purpose, methods, results, and conclusions | Abstract provides summary and written according to journal requirements |
| **Introduction** |  |
| ***Problem formulation*** - Description and significance of the problem/phenomenon studied; review of relevant theory and empirical work; problem statement | Methodological history and overview provided |
| ***Purpose or research question*** - Purpose of the study and specific objectives or questions | Research aim explicitly mentioned |
| **Methods** |  |
| ***Qualitative approach and research paradigm*** - Qualitative approach (e.g., ethnography, grounded theory, case study, phenomenology, narrative research) and guiding theory if appropriate; identifying the research paradigm (e.g., postpositivist, constructivist/ interpretivist) is also recommended; rationale** | Process of consultation described |
| ***Researcher characteristics and reflexivity*** - Researchers’ characteristics that may influence the research, including personal attributes, qualifications/experience, relationship with participants, assumptions, and/or presuppositions; potential or actual interaction between researchers’ characteristics and the research questions, approach, methods, results, and/or transferability | No qualitative analysis conducted |
| ***Context*** - Setting/site and salient contextual factors; rationale** | Contextual information not provided |
| ***Sampling strategy*** - How and why research participants, documents, or events were selected; criteria for deciding when no further sampling was necessary (e.g., sampling saturation); rationale** | Consultation groups described |
| ***Ethical issues pertaining to human subjects*** - Documentation of approval by an appropriate ethics review board and participant consent, or explanation for lack thereof; other confidentiality and data security issues | Ethics approval provided |
| ***Data collection methods*** - Types of data collected; details of data collection procedures including (as appropriate) start and stop dates of data collection and analysis, iterative process, triangulation of sources/methods, and modification of procedures in response to evolving study findings; rationale** | Consultation process described |
| ***Data collection instruments and technologies*** - Description of instruments (e.g., interview guides, questionnaires) and devices (e.g., audio recorders) used for data collection; if/how the instrument(s) changed over the course of the study | Online questionnaire provided in appendix |
| ***Units of study*** - Number and relevant characteristics of participants, documents, or events included in the study; level of participation (could be reported in results) | Number of participants and professional demographics completing survey reported |
| ***Data processing*** - Methods for processing data prior to and during analysis, including transcription, data entry, data management and security, verification of data integrity, data coding, and anonymization/de-identification of excerpts | No qualitative analysis conducted |
| ***Data analysis*** - Process by which inferences, themes, etc., were identified and developed, including the researchers involved in data analysis; usually references a specific paradigm or approach; rationale** | Not applicable |
| ***Techniques to enhance trustworthiness*** - Techniques to enhance trustworthiness and credibility of data analysis (e.g., member checking, audit trail, triangulation); rationale** | Not applicable |
| **Results/findings** |  |
| ***Synthesis and interpretation*** - Main findings (e.g., interpretations, inferences, and themes); might include development of a theory or model, or integration with prior research or theory | Recommendations and considerations proposed with rationale |
| ***Links to empirical data*** - Evidence (e.g., quotes, field notes, text excerpts, photographs) to substantiate analytic findings | Reference to literature on methods |
| **Discussion** |  |
| ***Integration with prior work, implications, transferability, and contribution(s) to the field*** - Short summary of main findings; explanation of how findings and conclusions connect to, support, elaborate on, or challenge conclusions of earlier scholarship; discussion of scope of application/generalizability; identification of unique contribution(s) to scholarship in a discipline or field | Short summary provided with reference to examples of other studies and how this proposal extends current work |
| ***Limitations*** - Trustworthiness and limitations of findings | Limitations acknowledged |
| **Other** |  |
| ***Conflicts of interest*** - Potential sources of influence or perceived influence on study conduct and conclusions; how these were managed | CoI stated |
| ***Funding*** - Sources of funding and other support; role of funders in data collection, interpretation, and reporting | Funding stated (no finding source) |
