# Supplementary material for: Could master protocols be adapted for effectiveness-implementation hybrid studies?
Source: BMC Med Res Methodol. 2025 Nov 18;25:258. doi: 10.1186/s12874-025-02684-1 (PMC12625322; doi:10.1186/s12874-025-02684-1)
Supplement: Supplementary file 3 — Supplementary Material 3. [file 12874_2025_2684_MOESM3_ESM.docx]

Questionnaire provided to contributors of the consultations:

| **Question** | **Response type** |
| --- | --- |
| *Name* | Open text |
| *Do you agree to be individually named in the acknowledgements section of any resulting publications?* | Yes/No |
| *How would you like to be named in acknowledgements (including pre-nominal, e.g. Dr)?* | Open text |
| *Would you like a summary of the consultation and draft manuscript for further comment?* | Yes/No |
| *Please provide your email address:* | Open text |
| *What is your professional background?* | Researcher / academic |
|  | Medical doctor |
|  | Allied Health practitioner |
|  | Peak/Advocacy organisation |
|  | Not from a formal research or health practitioner background |
|  | Other: open text |
| *How long have you worked in your current field?* | 0 to <5 years |
|  | 5 to <15 years |
|  | 15 to <25 years |
|  | 25 to <35 years |
|  | 35 to <45 years |
|  | 45 to <55 years |
|  | 55+ years |
| *What is your country of residence?* | Open text |
